# Unusual cutaneous adverse reaction to an erectile dysfunction drug: A case report

**DOI:** 10.1016/j.eucr.2023.102637

**Published:** 2023-12-09

**Authors:** Ivânia Soares, Inês P. Amaral, Madalena P. Correia, Paulo Filipe

**Affiliations:** a Department of Dermatology, Hospital de Santa Maria, Avenida Professor Egas Moniz MB, 1649-028, Lisboa, Portugal; b Dermatology Clinic, Faculdade de Medicina da Universidade de Lisboa, Avenida Professor Egas Moniz MB, 1649-028, Lisboa, Portugal

**Keywords:** Cutaneous adverse reaction, Tadalafil, AGEP

## Abstract

We presents an unique and significant case of Acute Generalized Exanthematous Pustulosis (AGEP) associated with the use of tadalafil, a phosphodiesterase type 5 inhibitor for erectile dysfunction. AGEP, typically linked to antibiotics and antiepileptics, had not been previously reported with tadaladil in the MEDLINE database. The case involved a 40-year-old male who developed an erythematous rash, fever and sterile pustules after starting tadalafil. A skin biopsy confirmed AGEP. Discontinuation of the drug and treatment with prednsiolone cleared the dermatosis. This case emphasizes the need for awareness among healthcare providers regarding this potential adverse reaction, considering the growing use of tadalafil.

## 1 Introduction

We write to present an unique and significant case of acute generalized exanthematous pustulosis associated with the administration of tadalafil, a phosphodiasterase type 5 inhibitor employed in the treatment of erectile dysfunction. AGEP is an infrequent cutaneous adverse event characterized by the rapid appearance of aseptic pustules on erythematous skin. While AGEP is typically linked to the consumption of antibiotics and antiepileptics^1^ our extensive review of the MEDLINE database did not uncover any previously documented cases of AGEP associated with tadalafil administration. Therefore, our case report represents, to the best of our knowledge the first reported incidence of AGEP associated with tadalafil use.

## 2 Case presentation

A 40-year-old Brazilian male patient, phototype IV, with no previous medical history, presented with an erythematous pruritic rash over his trunk, extremities and face, accompanied by fever. Initially the patient denied any recent drug intake during his visit to primary healthcare provider. However, due to progression of the dermatosis, he sought further medical assistance at our department. Eventually, the patient disclosed his recent initiation of tadalafil for the treatment of erectile dysfunction, with symptoms manifesting five days following its commencement. The patient denied any past ocurrences of joint pain or familial cutaneous disorders. Upon physical examination, multiple minute non-follicular sterile pustules were observed against a backdrop of diffusely reddned skin, encompassing the trunk, arms and intertriginous zones (Fig. 1 a), Fig. 1 b)). Notably, the mucous membranes were unaffected. Laboratory evaluation revealed an elevation in total leukocyte count with a prominent neutrophilic predominance. Viral and bacterial serologies where negative. Subsequently, a skin biopsy was performed showing spongiform subcorneal pustules and mixed dermal infiltrate with neutrophils and eosinophils (Fig. 2). According to the EUROSCAR study group scoring system, our patient achieved a score of 10, indicating a definitive diagnosis of AGEP.^2^ The patient's tadalafil usage was discontinued and treatment commenced with oral prednisolone at a daily dosage of 40 mg. The patient refused hospitalization and was managed on an outpatient basis, leading to progressive pustular desquamation and improvement of symptoms over subsequent days. Follow-up examination after two weeks demonstrated complete resolution of the rash.

**Fig. 1 a):**
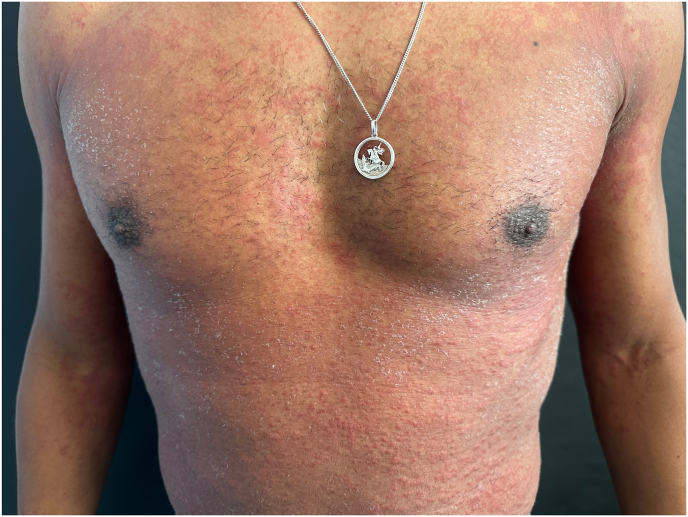
Erythematous and edematous plaques on the trunk.

**Fig. 1 b):**
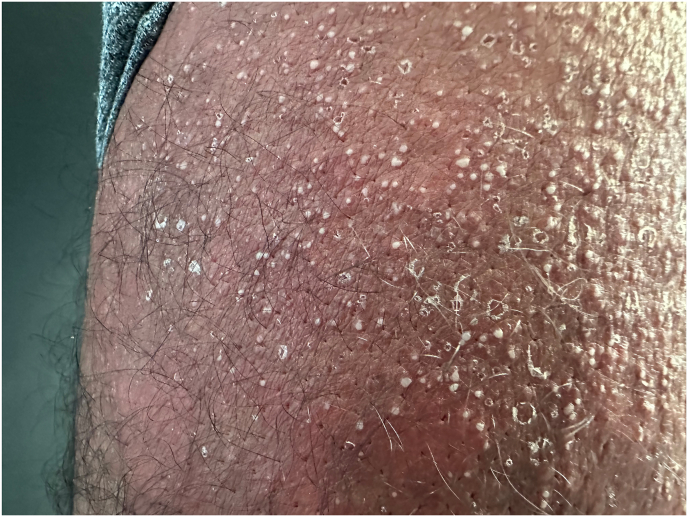
Numerous small, nonfolicullar pustules on erythematous plaques on the tighs.

**Fig. 2:**
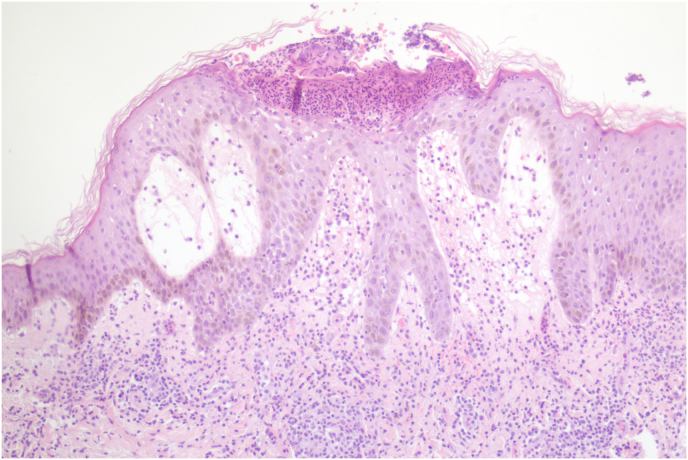
Spongiform subcorneal pustule and mixed dermal infiltrate with neutrophils and eosinophils (H&E x100).

## 3 Discussion

To ascertain the likelihood of a causal relationship between tadalafil and AGEP, we employed the Naranajo score, a validated tool for assessing adverse drug reactions. Our case yield a score of 5, indicating a probable association between tadalafil and AGEP development. Despite tadalafil being associated with some cutaneous adverse events such as urticaria, angioedema, Stevens-Johnson syndrome, and fixed druge eruptions no previous instances of AGEP have been documented.^3^ The precise mechanisms underlying AGEP in the context of tadalafil usage remains poorly understood; however an immune-mediated hypersensivity reaction can be involved as the remain culprit drugs of AGEP.^4^ This particular case underscores the necessity for healthcare providers to be cognizant of this infrequent but consequential potential adverse reaction that may manifest following tadalafil administration, especially given the escalating demand for erectile dysfunction medications, particularly tadalafil. This distinctive medication, with a prolonged plasma half-time of 17.5 hours, confers the ability to augment penile blood flow over 36-h period, endowing it with endearing sobiquet of “le weekend” among the French and making it appealing between male patients.^3^

## 4 Conclusion

Prompt recognition of AGEP, cessation of the causative agent and appropriate therapeutic intervention can yield a favorable prognosis for affected patients. Further investigation is warrented to elucidate the intricate mechanisms linking AGEP and tadalafil usage and to establish optimal management strategies for individuals experiencing this adverse drug reaction.

## Funding

This research did not receive any specific grant from funding agencies in the public, commercial, or not-for-profit sectors.

## Author's contributions

1. Ivânia Soares- Conceptualization, Methodology, Roles/Writing - original draft.

2. Inês Pereira Amaral- Roles/Writing - original draft.

3. Madalena Pupo Correia- Writing - review & editing.

4. Paulo Filipe- Writing - review & editing.

## Declaration of competing interest

None.
